# Evaluation of blood impurity removal efficiency using the QuEChERS method

**DOI:** 10.1007/s11419-025-00740-5

**Published:** 2025-10-06

**Authors:** Haruki Kuze, Haruhi Yoshida, Hikaru Tamagawa, Taichi Nishihori, Yuri Tokugawa, Fumika Yamamoto, Hiroshi Matsumoto, Kazuo Harada

**Affiliations:** 1 School of Pharmaceutical Sciences, The University of Osaka, Yamadaoka 1-6, Suita, Osaka 565-0871 Japan; 2 Department of Legal Medicine, Graduate School of Medicine, The University of Osaka, Yamadaoka 2-2, Suita, Osaka 565-0871 Japan; 3 Education and Research Institute for Death Control and Prevention, Graduate School of Medicine, The University of Osaka, Yamadaoka 2-2, Suita, Osaka 565-0871 Japan

**Keywords:** Impurity, Deproteinization, QuEChERS, Recovery rate, Matrix factor

## Abstract

**Purpose:**

Comparison of the impurity removal efficiencies of the deproteinization and Quick, Easy, Cheap, Effective, Rugged, Safe (QuEChERS) methods, which are pretreatment methods for drug analysis adopted by many forensic autopsy institutions, was performed.

**Method:**

Residual cardiac blood samples were pretreated using deproteinization and QuEChERS methods. The residual amounts of total proteins, total lipids, glucose, galactose, electrolytes, and inorganic elements were measured. We also compared the recovery rates and matrix factors when using liquid chromatography/tandem mass spectrometry (LC/MS/MS).

**Results:**

The residual rates of total proteins, total lipids, glucose, galactose, and electrolytes using the deproteinization method were 16%, 75%, 75%, 90%, and 91%, respectively. In contrast, the QuEChERS method showed 1.1%, 11%, 7.6%, 9.4%, and 20%, respectively. The amounts of Mg and Mn in QuEChERS increased compared with those before treatment, but other inorganic elements remained at 9.6–89% during deproteinization and 0.30–17% in the QuEChERS. The recovery rate of metformin was low in QuEChERS; however, no differences were observed in the recovery rates or matrix factors of the other 16 drugs between deproteinization and QuEChERS.

**Conclusions:**

This study quantitatively demonstrated that QuEChERS is extremely efficient at removing impurities from blood compared with deproteinization methods. QuEChERS has poor recovery rates for highly polar drugs but does not prevent their detection. The QuEChERS method is superior to the deproteinization method, considering the load of impurities on the analytical instruments.

**Supplementary Information:**

The online version contains supplementary material available at 10.1007/s11419-025-00740-5.

## Introduction

Gas chromatography/mass spectrometry (GC/MS) and liquid chromatography/mass spectrometry (LC/MS), which have excellent specificity and sensitivity, are commonly used for drug analysis in forensic autopsies [1–9]. If a biological sample, such as blood, is directly injected into these analyses, impurities such as proteins, lipids, carbohydrates, and metals contained in the sample can damage the instruments, reduce their sensitivity, and cause fluctuations in the quantitative values due to matrix effects [10–13]. To prevent these problems, it is necessary to remove impurities from the sample. The methods used for drug analysis in forensic autopsies include liquid–liquid partitioning using non-polar organic solvents, solid-phase extraction using mini-columns, deproteinization using water-soluble organic solvents, and the Quick, Easy, Cheap, Effective, Rugged, Safe (QuEChERS) method, which uses MgSO_4_ to separate acetonitrile and water and allows for liquid–liquid partitioning. These methods may also be combined with dispersive solid-phase extraction, in which particles surface-modified with octadecyl silyl (C18) or primary-secondary amine are added to the extraction solution. Taking into consideration the comprehensiveness of the drugs to be detected, throughput, and other institutional factors, we considered deproteinization and QuEChERS to be appropriate pre-treatment methods [14–16]. However, the impurity removal rates of these pretreatment methods have not been quantitatively evaluated. Therefore, we compared the impurity removal efficiency in blood samples between the deproteinization and QuEChERS methods and further validated the QuEChERS method.

## Materials and methods

### Reagents

LC/MS-grade ultrapure water, methanol, and acetonitrile were purchased from FUJIFILM Wako Pure Chemical Corporation (Osaka, Japan). Methoxyamine hydrochloride and *N*-methyl-(*N*-trimethylsilyl) trifluoroacetamide for the derivatization of glucose and galactose were purchased from Sigma-Aldrich (Merck KGaA, Darmstadt, Germany) and GL Sciences (Tokyo, Japan), respectively. The standard chemicals used for validation were purchased from FUJIFILM Wako Pure Chemical Corporation and Sigma-Aldrich, and 1 mg/mL methanol solutions were prepared as stock solutions and diluted appropriately.

### Sample preparation

The samples were obtained from the right heart blood samples from the Department of Legal Medicine, Osaka University, where no drugs were detected. To evaluate the individual differences in impurities, matrix factors, and recovery rates, each individual sample was used without mixing. For deproteinization, 450 μL of ultrapure water was added to 50 μL of sample, followed by 500 μL of acetonitrile. After vortexing and centrifuging at 10,000 × *g* for 3 min, supernatants were collected. The supernatant was dried using a centrifugal concentrator, and the residue was dissolved in 50 μL of ultrapure water. For the QuEChERS method, a kit Q-Sep Q251 manufactured by RESTEK (Bellefonte, PA, USA), in which MgSO_4_, C18, and primary-secondary amines were pre-aliquoted in tubes, was used. The sample (50 μL) was added to the tube, followed by 450 μL of ultrapure water, 500 μL of acetonitrile, and a 4-mm stainless steel bead. After vortexing and centrifuging at 10,000 × *g* for 3 min, supernatants were collected. The supernatant was dried using a centrifugal concentrator (CVE-3110, TOKYO RIKAKIKAI CO., LTD., Tokyo, Japan), and the residue was dissolved in 50 μL of ultrapure water. To analyze the impurity removal rate, in addition to the samples prepared using deproteinization and QuEChERS methods as described above, 50 μL of untreated blood was used as a reference.

### Total protein and total lipid measurement

The total protein and lipid contents in the samples were quantified using the Fuji Dry Chem slide, TP-PIII, and TG-PIII (provided by FUJIFILM) on a Fuji Dry Chem (7000 V, FUJIFILM) system.

### Electrolyte and metal element measurement

To measure the electrolytes, 50 μL of the sample solution was diluted with 950 μL of ultrapure water and measured using a portable conductivity meter (LAQUAtwin-EC-33, HORIBA, Kyoto, Japan).

Metal elements were analyzed as described by Cesbron et al. [17]. The sample solution (50 μL) was diluted with 4.95 mL of a solution containing 1% (v/v) nitric acid, 0.5% (v/v) 1-butanol, 0.1% (w/v) Triton X-100, and 1 mg/L gallium, and subjected to inductively coupled plasma-mass spectrometry (ICP-MS). An Agilent 7700x instrument (Agilent Technologies, Santa Clara, CA, USA) was used for the ICP-MS measurements. The RF power was set to 1550 W. Argon and helium were used as the carrier and collision gases, respectively. The carrier gas was 1.05 L/min. The *m/z* scan ranges were 2–15, 20–39, and 42–260. The integration time was 0.1 s for each peak point and 0.6 s for each mass number. Tuning was conducted by tuning the mix containing 1 μg/L Li, Co, Y, Ce, and Tl in 5% nitric acid (Agilent Technologies) immediately before measuring the samples.

### GC/MS for glucose and galactose measurements

GC/MS for glucose and galactose measurements was modified from the method described by Chan et al. [18]. The blood sample (50 μL) was transferred to a 1.5 mL microtube, and 13 μL of 1 mM ribitol in methanol was added as an internal standard. Subsequently, 300 μL of methanol was added, and the mixture was vortexed for 15 s. After centrifugation at 15,000 × *g* for 15 min, 250 μL of the supernatant was transferred to a 2.0 mL Eppendorf microtube and dried overnight using a centrifugal concentrator. A total of 50 μL of 1 mg/mL methoxyamine in pyridine was added to the dry residue, and methoxylation was performed using a ThermoMixer (Eppendorf, Hamburg, Germany) by shaking at 80 °C for 15 min at 1200 rpm. After adding 50 μL of *N*-methyl-*N*-(trimethylsilyl)trifluoroacetamide was performed in ThermoMixer by shaking at 80 °C for 15 min at 1200 rpm. After cooling for 5 min, the sample was centrifuged at 15,000 × *g* for 15 min, and 100 μL of the supernatant was transferred into sample vials for GC/MS. GC/MS was performed using a GCMS-QP2010 SE instrument (Shimadzu Corp., Kyoto, Japan). The autosampler was an AOC-20i/s (Shimadzu Corp.), and the column was a GC column BPX5 (0.25 mm i.d. × 30 m, 0.25 μm thick, Trajan, Melbourne, Australia). Helium was used as the carrier gas, and the flow rate was controlled to maintain the column linear velocity at 39.0 cm/s. The splitting ratio was 1:30. The sample injection volume was 1 μL. The temperature of the vaporization chamber was set to 250 °C. The temperature of the column oven was held at 60 °C for 2 min, raised to 330 °C at a rate of 15 °C/min, and held there for 3.34 min. Sample ionization was performed using the electron ionization method. The temperature at the ion source was set at 200 °C, and that at the interface section was set at 280 °C. The detector voltage was tuned 3 min after sample injection, and the scan data were collected from 3.5 to 23.14 min. The scan time was 0.20 s. The detector voltage was set at + 0.1 kV from the tuning value, and the scan range was set to *m/z* 45–600.

### LC/MS

LC/MS was performed using an LCMS8060 instrument (Shimadzu Corp.). LC separations were conducted at 40 °C with a Kinetex XB-C18 (2.6 μm, 2.1 mm i.d. × 100 mm, Phenomenex, Torrance, CA, USA). The mobile phase was delivered at a flow rate of 0.3 mL/min using a gradient elution profile consisting of Solvent A (0.1% formic acid and 10 mmol/L ammonium formate in distilled water) and Solvent B (0.1% formic acid and 10 mmol/L ammonium formate in methanol). The initial composition of the binary solvent was 5% Solvent B, which was increased to 95% at 7.5 min. The composition of Solvent B was maintained at 95% for 2.5 min. The injection volume was 5 μL. The mass spectrometer was operated with a DUIS™ source in positive ion mode and set in the multiple reaction monitoring (MRM) mode. Nebulizer, heating, and drying gas flows were set to 2, 10, 10 L/min, respectively, whereas interface, desolvation line, and heat block temperatures were set at, 300, 250, and 400 °C, respectively. The dwell time for each MRM transition was set to 2 ms. The MRM transitions are shown in Supplemental Table 1.

### Validation

Blood samples were prepared using deproteinization or the QuEChERS method, followed by LC/MS measurements. Based on the calibration curve obtained using a dilution series by preparing the standard stock solution with ultrapure water and methanol, concentration value A was obtained by adding the standard chemicals to the blood samples before pretreatment, and concentration value B was obtained by adding standard chemicals to the blood samples after pretreatment. The matrix factor (MF) value was calculated as the ratio of B/(added concentration), and the recovery rate was calculated as A/B × 100(%). With the exception of etizolam and temazepam, the added concentration was 1000 ng/mL. Because the signals for etizolam and temazepam were saturated at 1000 ng/mL, these were evaluated at 316 ng/mL.

The accuracy and precision of the QuEChERS method were evaluated. Standard chemicals were added to the blood at four concentrations: near the lower limit of quantification, low, medium, and high concentrations, in the concentration range of 1–1000 ng/mL. Measurements were repeated five times on each analysis day over three days, and intraday and interday variations were calculated. Accuracy value was defined as the degree of agreement between the average of the measured concentrations of 15 times and the concentration of the standard solution added. As internal standards, stable isotope-labeled compounds 7-aminoflunitrazepam-d_7_, acetaminophen-d_3_, alprazolam-d_5_, diazepam-d_5_, etizolam-d_3_, metformin-d_6_, and zolpidem-d_7_ were used for each non-label compound measurement. For other chemicals, diazepam-d_5_ was used as an internal standard.

## Results

### Impurity removal rate of deproteinization and QuEChERS methods

**Fig. 1:**
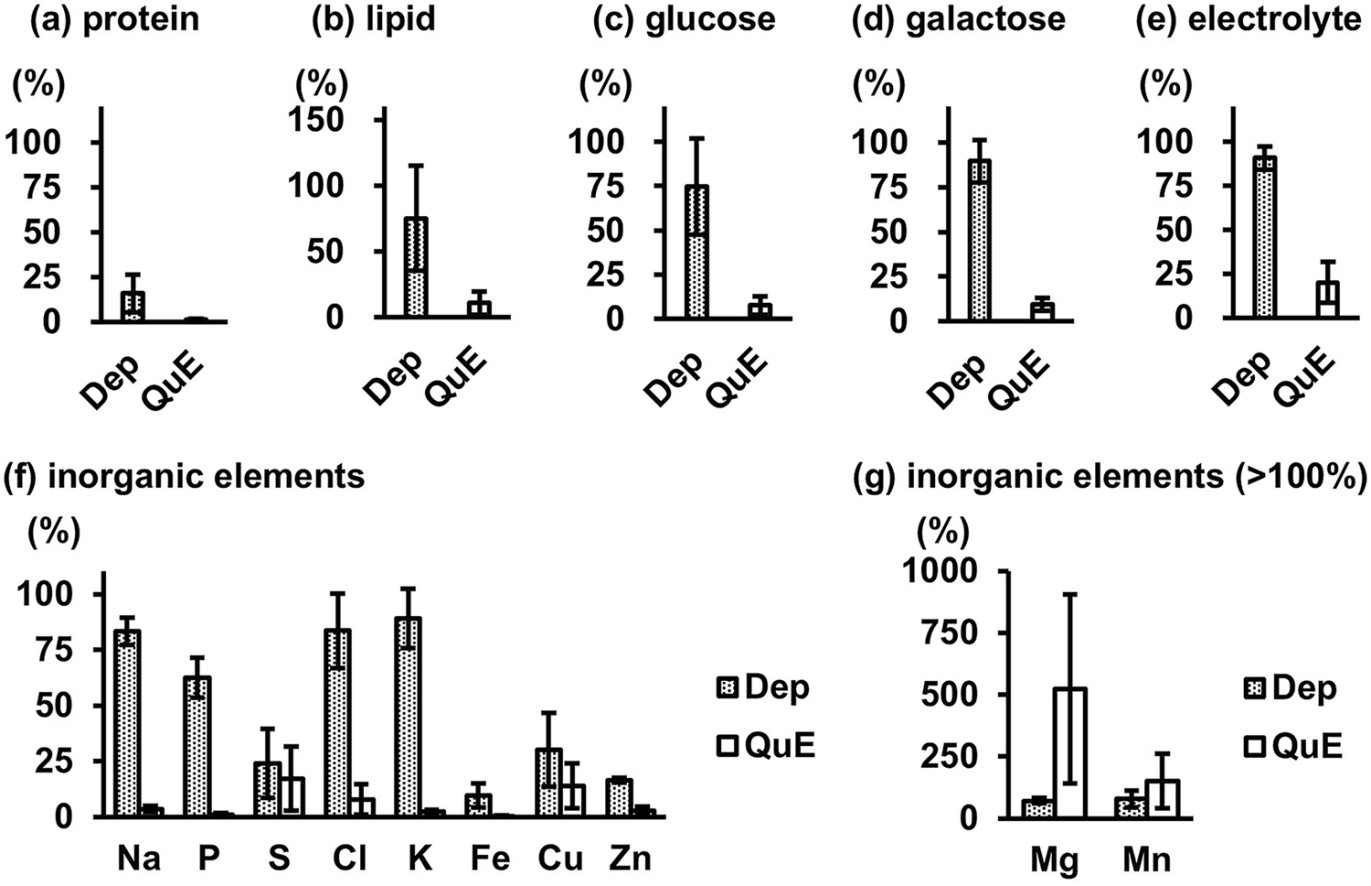
Residual amounts of contaminants in cardiac blood samples after deproteinization (Dep) and QuEChERS (QuE) treatment. sThe contaminants analyzed were **a** total proteins, **b** total lipids, **c** glucose, **d** galactose, **e** electrolyte, and **f**, **g** inorganic elements. The values are shown as relative values, with the content of each contaminant in the untreated blood sample considered 100%. Blood samples were obtained from six cadavers. Bars and error bars represent means and standard deviations, respectively

Right heart blood samples obtained from six cadavers were treated using deproteinization and QuEChERS methods, and the residual amounts of contaminants, including total proteins, total lipids, glucose, galactose, and electrolytes, were measured (Fig. 1). The remaining total proteins, relative to the amount contained in the blood before pretreatment, was 16% using the deproteinization method and 1.1% using the QuEChERS method (Fig. 1a). The residual rates of total lipids, glucose, galactose, and electrolytes using the deproteinization method were 75%, 75%, 90%, and 91%, respectively. In contrast, the QuEChERS method showed 11%, 7.6%, 9.4%, and 20%, respectively (Fig. 1b–e). Metal element measurements by ICP-MS showed that the residual rates of sodium, potassium, phosphorus, and chlorine were significantly lower using the QuEChERS method than using the deproteinization method (Fig. 1f). Magnesium and manganese increased with QuEChERS compared with the sample before pretreatment (Fig. 1g).

### Recovery and MF of deproteinization and QuEChERS methods

The recovery rates and MF values of 17 drugs obtained using the deproteinization and QuEChERS methods are shown in Table 1. The selection of these drugs was based on their relatively high frequency of detection in our laboratory and the fact that we had standard substances of these drugs. The concentration of the analytes was set to 316–1000 ng/mL, which is the upper limit of quantification at which carryover and saturation do not occur. The recovery rate of all compounds was higher using the deproteinization method. In particular, metformin showed a large difference, with 110% with deproteinization and 36% with QuEChERS. In contrast, the MF values were similar for both methods, and no differences were observed.

**Table 1:** Recovery and matrix factor of deproteinization and QuEChERS

	Recovery (%)		Matrix factor (%)	
	Deproteinized	QuEChERS	Deproteinized	QuEChERS
7-aminoflunitrazepam	102 ± 13	78 ± 5	92 ± 14	100 ± 19
Acetaminophen	96 ± 10	82 ± 8	105 ± 22	96 ± 30
Alprazolam	103 ± 15	74 ± 5	92 ± 11	84 ± 45
Diazepam	103 ± 15	74 ± 5	93 ± 15	101 ± 22
Ephedrine	98 ± 11	65 ± 3	92 ± 15	96 ± 19
Etizolam	103 ± 16	67 ± 5	81 ± 11	104 ± 21
Flunitrazepam	106 ± 16	77 ± 5	87 ± 12	91 ± 21
Lacosamide	101 ± 14	82 ± 5	96 ± 13	97 ± 17
Metformin	111 ± 9	37 ± 10	13 ± 5	13 ± 4
Methylphenidate	101 ± 15	63 ± 4	97 ± 14	105 ± 17
Mirtazapine	105 ± 16	45 ± 2	92 ± 14	103 ± 20
Nifedipine	104 ± 17	77 ± 5	98 ± 16	122 ± 22
Sulpiride	99 ± 13	72 ± 6	104 ± 15	114 ± 16
Temazepam	102 ± 15	64 ± 3	98 ± 11	98 ± 21
Trazodone	101 ± 16	65 ± 4	97 ± 14	102 ± 21
Warfarin	99 ± 13	78 ± 5	109 ± 17	108 ± 14
Zolpidem	102 ± 16	69 ± 4	98 ± 14	105 ± 21

The values are means ± standard deviation obtained using right heart blood samples from six individuals. The added concentration was 1000 ng/mL except for etizolam and temazepam. Because the signals for etizolam and temazepam were saturated at 1000 ng/mL, they were evaluated at 316 ng/mL

### Precision and accuracy of the QuEChERS method

The results of the evaluation of the precision and accuracy of 17 drugs are shown in Table 2. The intraday and interday variations were 3–31% and 5–32%, respectively. The accuracy values ranged from 86 to 140%. However, for many items, the intraday and interday variations were less than 15% (20% at the limit of quantitation (LOQ)), and the accuracy values were within the range of 100 ± 15% (100 ± 20% at LOQ).

**Table 2:** Precision and accuracy of the QuEChERS method

	Conc. (ng/mL)				Intraday precision (RSD%)				Interday precision (RSD%)				Accuracy (%)			
	LOQ	Low	Middle	High	LOQ	Low	Middle	High	LOQ	Low	Middle	High	LOQ	Low	Middle	High
7-Aminoflunitrazepam*	3.2	10	100	1000	14	9	3	7	15	13	6	9	98	98	96	97
Acetaminophen*	32	100	316	1000	23	8	7	11	25	11	8	12	97	94	94	93
Alprazolam*	1.0	3.2	32	1000	22	14	6	8	23	16	10	8	97	96	98	96
Diazepam*	1.0	3.2	32	1000	22	12	11	11	32	12	14	12	108	105	98	97
Ephedrine	3.2	10	32	1000	17	10	7	12	18	16	10	16	114	100	94	105
Etizolam*	3.2	10	32	316	12	12	8	4	13	17	9	5	100	115	112	89
Flunitrazepam	1.0	10	100	1000	15	14	10	11	20	17	11	13	93	98	96	98
Lacosamide	1.0	3.2	100	1000	17	12	5	11	18	12	6	15	103	99	96	100
Metformin	10	32	100	1000	11	8	4	10	20	12	5	11	99	96	95	94
Methylphenidate	1.0	3.2	100	1000	9	9	7	13	15	10	12	14	121	102	94	107
Mirtazapine	1.0	3.2	100	1000	12	14	9	14	14	14	14	14	140	111	95	93
Nifedipine	3.2	10	100	1000	22	10	9	12	23	12	10	14	113	101	100	102
Sulpiride	1.0	3.2	100	1000	12	10	13	16	12	10	14	18	126	102	86	99
Temazepam	1.0	3.2	10	316	20	14	10	6	25	18	13	7	97	113	115	101
Trazodone	1.0	3.2	100	1000	16	10	5	11	17	12	10	13	121	104	93	98
Warfarin	1.0	3.2	100	1000	25	17	5	12	28	18	9	14	87	87	99	94
Zolpidem*	1.0	3.2	100	1000	31	15	3	8	32	16	9	9	99	94	95	96

The standard chemicals were added to the blood at four concentrations: near the lower limit of quantification (LOQ), and low, medium, and high concentrations, in the concentration range of 1–1000 ng/mL. Measurements were repeated five times on each analysis day over three days, and intraday and interday variations were calculated. Accuracy value was defined as the degree of agreement between the average of the measured concentrations of a total of 15 times and the concentration of the standard solution added

^*^: Stable isotope-labeled compounds were used as internal standards. For other chemicals, diazepam-d_5_ was used as an internal standard

## Discussion

Proteins, lipids, carbohydrates, and metal elements contained in biological samples are impurities in GC/MS and LC/MS measurements. These impurities lead to clogging of the mobile phase flow line, ionization probe, and ion inlet, reduction in column resolution due to adsorption, and decrease in detection sensitivity due to ionization suppression [10–13, 19, 20]. For all these impurities, the residual amount in the sample was lower with the QuEChERS method than with the deproteinization method, indicating a higher removal efficiency. Although approximately 85% of the total proteins was removed using the deproteinization method, only approximately 99% was removed using the QuEChERS method. Although only approximately 10–32% of lipids, glucose, galactose, and electrolytes were removed using the deproteinization method, approximately 80–92% were removed using the QuEChERS method, showing a large difference in removal efficiency. When the residual amounts of each metal element in the sample were evaluated, the amounts of Mg and Mn were higher in the QuEChERS method than in the deproteinization method. This was thought to be largely due to the influence of MgSO_4_ and the adsorbents included in the QuEChERS kit. However, as mentioned above, the total amount of electrolytes was more efficiently removed by the QuEChERS method than by the deproteinization method, and the increase in Mg and Mn was much smaller than the decrease in the total amount of electrolytes. The data presented in this study, which quantitatively evaluate the impurity removal rate by pretreatment, are extremely valuable.

Although when using the two methods, we obtained a similar MF value, which influences quantification, recovery rates tended to be higher when using the deproteinization method. Notably, there was a particularly large difference between the deproteinization and QuEChERS methods with respect to the rate at which metformin was recovered. Metformin has an extremely high water solubility, with an octanol/water partition ratio (LogP_ow_) of −2.64 (Supplemental Table 1), and should accordingly be predominantly distributed in the aqueous layer, even when using the QuEChERS method. Furthermore, as blood impurities, carbohydrates and electrolytes should similarly be distributed within the aqueous layer when using QuEChERS. Consequently, a high correlation between the recovery of metformin and the residual fractions of carbohydrates and electrolytes was anticipated, which was indeed the result obtained. Contrastingly, the LogP_ow_ values obtained for the other assessed compounds were all greater than 0, implying a predominant distribution in the acetonitrile layer. Indeed, using QuEChERS method, with the exception of mirtazapine, for which we obtained a recovery of approximately 50%. The rates at which all the other assessed compound were recovered exceeded 50%. Notably, these recoveries did not appear to be substantially influenced by the removal of impurities. In addition, a low rate of recovery indicates that a target compound may have been overlooked at low concentrations. Thus, deproteinization was superior to QuEChERS in terms of the comprehensiveness of detection. However, by selecting appropriate internal standards for analytes with low recovery rates, the QuEChERS method achieved good results from the perspectives of accuracy and precision.

In this study, no correlation was found between the removal efficiency of the contaminants and the MF values or analyte recovery rates. However, there is no doubt that the deproteinization method left more impurities, which were then introduced into the LC/MS device. This suggests that the degree of equipment contamination may progress more rapidly with deproteinization, leading to more frequent maintenance. Since adopting this method, we have been performing analyses at a rate of 100 to 200 injections per month over the past 2 years, during which time the frequency of mechanical problems in the Department of Legal Medicine has been substantially reduced, and no maintenance of the mass spectrometer has been required.

## Conclusions

This is the first study to compare the impurity removal efficiencies of pretreatment methods for drug analysis in legal autopsies. The QuEChERS method removed more impurities than the deproteinization method. Considering that drug analysis will be conducted over the long term, the QuEChERS method may be superior because such impurities accumulate on the analytical equipment.

## Supplementary Information

Below is the link to the electronic supplementary material.

Supplementary file1 (DOCX 26 KB)

## Data Availability

The datasets generated during the current study are available from the corresponding author upon reasonable request.
