# Supplementary material for: Evaluation of blood impurity removal efficiency using the QuEChERS method
Source: Forensic Toxicol. 2025 Oct 6;44(1):217–23. doi: 10.1007/s11419-025-00740-5 (PMC12858495; doi:10.1007/s11419-025-00740-5)
Supplement: Supplementary file 1 — Supplementary file1 (DOCX 26 KB) [file 11419_2025_740_MOESM1_ESM.docx]

**Supplemental Table 1 MRM transition, retention time and octanol/water partition ratio of target analytes in this study**

|  | quantitative ion | confirmation　ion | retention  time (min) | LogP_ow_ |
| --- | --- | --- | --- | --- |
| 7-aminoflunitrazepam | 284.10>135.10 | 284.10>226.15 | 4.73 | 1.46 [1] |
| acetaminophen | 152.00>110.00 | 152.00>65.05 | 2.62 | 0.46 [2] |
| alprazolam | 309.10>281.10 | 309.10>205.10 | 6.42 | 2.09 [3] |
| diazepam | 285.10>193.05 | 285.10>154.10 | 6.88 | 2.82 [2, 4] |
| ephedrine | 166.10>148.05 | 166.10>117.05 | 2.89 | 0.93 [5] |
| etizolam | 343.05>314.10 | 343.05>138.15 | 6.59 | 2.87 [1] |
| flunitrazepam | 314.05>268.15 | 314.05>239.25 | 6.07 | 2.06 [6] |
| lacosamide | 251.00>108.10 | 251.00>91.00 | 4.36 | 0.83 [1] |
| metformin | 130.10>60.00 | 130.10>71.00 | 0.84 | -2.64 [5, 6] |
| methylphenidate | 234.15>84.05 | 234.15>56.05 | 4.44 | 2.35 [1] |
| mirtazapine | 266.10>195.05 | 266.10>72.10 | 4.56 | 2.81 [7] |
| nifedipine | 347.00>315.05 | 347.00>254.10 | 6.25 | 2.2 [8] |
| sulpiride | 342.15>112.15 | 342.15>58.10 | 2.48 | 0.57 [4] |
| temazepam | 301.05>255.10 | 301.05>283.15 | 6.49 | 2.2 [9] |
| trazodone | 372.15>176.10 | 372.15>148.05 | 4.99 | 3.85 [7] |
| warfarin | 309.15>163.05 | 309.15>251.05 | 6.87 | 2.60 [4] |
| zolpidem | 308.15>235.20 | 308.15>236.20 | 4.73 | 1.20 [10] |
| 7-aminoflunitrazepam-d_7_ | 291.00>138.10 | 291.00>230.10 | 4.73 |  |
| acetaminophen-d_4_ | 156.00>114.10 | 156.00>69.10 | 2.62 |  |
| alprazolam-d_5_ | 314.00>286.05 | 314.00>210.10 | 6.42 |  |
| diazepam-d_5_ | 290.15>154.05 | 290.15>198.20 | 6.88 |  |
| etizolam-d_3_ | 346.00>317.05 | 346.00>138.10 | 6.59 |  |
| metformin-d_6_ | 136.00>60.05 | 136.00>77.10 | 0.84 |  |
| zolpidem-d_7_ | 315.00>242.15 | 315.00>243.15 | 4.73 |  |

Reference

1. CAS SciFinder® (Access on 7^th^ Aug. 2025)
2. Vuckovic D, Cudjoe E, Hein D, Pawliszyn J (2008) Automation of solid-phase microextraction in high-throughput format and applications to drug analysis. Anal Chem 80(18): 6870-6880. <https://doi.org/10.1021/ac800936r>
3. Fenneteau F, Poulin P, Nekka F (2010) Physiologically based predictions of the impact of inhibition of intestinal and hepatic metabolism on human pharmacokinetics of CYP3A substrates. J Pharm Sci 99(1): 486-514. <https://doi.org/10.1002/jps.21802>
4. Hughes LD, Palmer DS, Nigsch F, Mitchell JBO (2008) Why are some properties more difficult to predict than others? A study of QSPR models of solubility, melting point, and Log P. J Chem Inf Model 48(1): 220-232. <https://doi.org/10.1021/ci700307p>
5. Ottaviani G, Martel S, Carrupt PA (2006) Parallel artificial membrane permeability assay: a new membrane for the fast prediction of passive human skin permeability. J Med Chem 49(13): 3948-3954. <https://doi.org/10.1021/jm06230>+
6. Sanderson H, Thomsen M (2009) Comparative analysis of pharmaceuticals versus industrial chemicals acute aquatic toxicity classification according to the United Nations classification system for chemicals. Assessment of the (Q)SAR predictability of pharmaceuticals acute aquatic toxicity and their predominant acute toxic mode-of-action. Toxicol Lett 187(2): 84-93. <https://doi.org/10.1016/j.toxlet.2009.02.003>
7. Wan H, Ahman M, Holmén AG (2009) Relationship between brain tissue partitioning and microemulsion retention factors of CNS drugs. J Med Chem 52(6): 1693-1700. <https://doi.org/10.1021/jm801441s>
8. Yu Z, Peldszus S, Huck PM (2007) Optimizing gas chromatographic-mass spectrometric analysis of selected pharmaceuticals and endocrine-disrupting substances in water using factorial experimental design. J Chromatogr A 1148(1): 65-77. <https://doi.org/10.1016/j.chroma.2007.02.047>
9. Hummel D, Löffler D, Fink G, Ternes TA (2006) Simultaneous determination of psychoactive drugs and their metabolites in aqueous matrices by liquid chromatography mass spectrometry. Environ Sci Technol 40(23): 7321-7328. <https://doi.org/10.1021/es061740w>
10. Plumb RS, Potts WB 3^rd^, Rainville PD, Alden PG, Shave DH, Baynham G, Mazzeo JR (2008) Addressing the analytical throughput challenges in ADME screening using rapid ultra-performance liquid chromatography/tandem mass spectrometry methodologies. Rapid Commun Mass Spectrom 22(14): 2139-2152. <https://doi.org/10.1002/rcm.3594>
